# Heat Shock Proteins 60 and 70, Ki67 and Caspase 3 Are Differentially Expressed in the Canine Pregnant and Non-Pregnant Uterus and Ovaries

**DOI:** 10.3390/vetsci13050482

**Published:** 2026-05-16

**Authors:** Schäfer-Somi Sabine, Binli Firdevs, Kaya Duygu, Karadag Muhammed Ali, Ay Serhan, Findik Murat, Aslan Selim

**Affiliations:** 1 Department of Small Animals and Horses, Clinical Center for Reproduction, University of Veterinary Medicine Vienna, 1210 Vienna, Austria; 2 Department of Obstetrics and Gynecology, Faculty of Veterinary Medicine, Ondokuz Mayıs University, 55139 Samsun, Türkiye; firdevsbinli@gmail.com (B.F.); serhan.ay@omu.edu.tr (A.S.); mfindik@omu.edu.tr (F.M.); 3 Department of Obstetrics and Gynecology, Faculty of Veterinary Medicine, Dokuz Eylül University, Kiraz, 35210 İzmir, Türkiye; kaya.duygu@deu.edu.tr; 4 Department of Obstetrics and Gynecology, Faculty of Veterinary Medicine, Kafkas University, 36100 Kars, Türkiye; muhammetali.karadag@deu.edu.tr; 5 Department of Obstetrics and Gynecology, Faculty of Veterinary Medicine, Near East University, 99138 Nicosia, Cyprus; selim.aslan@neu.edu.tr

**Keywords:** apoptosis, cellular stress, dogs, heat shock proteins, pregnancy

## Abstract

Heat shock proteins (HSPs) fulfil protective tasks in the whole organism; in pregnant dogs, they are expressed in the ovary, placenta and preimplantation embryo. Our objective was to compare the expression of HSP60 and -70, along with indicators of proliferation and apoptosis, in the non-pregnant and pregnant uterus/placenta and ovaries. Tissues were obtained after ovariohysterectomy and examined by means of immunohistochemistry. There were differences between pregnant and non-pregnant tissues: the expression level of HSP70 during preimplantation in superficial cells was significantly lower than that in early diestrus, with similar results observed for Ki67. The immunosignal for HSP70 was significantly decreased during the postimplantation stage in almost all cell types, whilst the number of HSP60-positive cells did not change. In pregnant animals, the number of Ki67-positive cells significantly increased until the postimplantation stage. In the placenta and trophoblast, the expression of HSP60 and -70 was strong, while no HSP70 signal was detected in endometrial epithelial cells. The caspase 3 immunosignal in the uterus and placenta was generally weak. In the corpora lutea, HSP60, HSP70 and caspase 3 were mainly detected in theca lutein cells, while no signal for KI67 was seen. In follicles, caspase 3 and KI67 expression was low, except in granulosa cells of tertiary follicles and oocytes. We conclude that the different expression of HSPs in pregnant and non-pregnant animals may point towards different regulatory and/or protective tasks.

## Simple Summary

This study provides a basis concerning the distribution of heat shock proteins (HSPs) inside the pregnant canine uterus. In dogs, the establishment of pregnancy requires a balance between proliferative mechanisms, initiated by the embryo, and apoptosis inside maternal tissue, both enabling invasive growth. HSP60 and -70 contribute to the regulation of invasive cell growth and were detected in canine uterine and embryo tissue and in serum. As a next step, the distribution of these cellular stress proteins, together with further indicators of proliferation and apoptosis (Ki67 and caspase 3), was examined inside the pregnant uterus and ovaries during the pre- and postimplantation stages and compared with that in tissue from non-pregnant dogs. Importantly, HSP70 expression in superficial cells was significantly decreased in comparison with that in non-pregnant animals. In pregnant animals, while the number of HSP60-positive cells stayed equal, the number of cells positive for HSP70 further decreased during the postimplantation stage in almost all cell types; this might mirror the previous need for regulatory anti-apoptotic mechanisms during implantation, no longer needed after decidualization.

## 1. Introduction

Heat shock proteins (HSPs) are cellular stress proteins that are ubiquitously expressed and are essential for the maintenance of cellular homeostasis [1]. Heat shock proteins are chaperones and mostly fulfil anti-apoptotic and protective roles within organisms [2]. They contribute to thermotolerance, stress tolerance, cell proliferation, embryogenesis and drug resistance. Furthermore, HSPs participate in cells’ physiological metabolic processes [3,4]. Some HSPs regulate the function and transcriptional activation of steroid hormone receptors [5]. Chaperones exert their effects either via a shield over hydrophobic regions of the DNA (HSP70) or by providing shelter for undisturbed DNA folding (chaperonins) [3]. Thereby, HSPs bind polypeptides and support and stabilize protein folding. However, some apoptosis-inhibitory HSPs such as HSP70 and -27 may contribute to cancer development [2]. Extracellular HSPs have been detected in exosomes and are supposed to contribute to cell–cell communication under physiological conditions but also under pathological conditions such as cancer [6]. Heat shock proteins can be grouped according to their molecular weight into high- and low-molecular-weight proteins. The high-molecular-weight HSPs comprise three families: HSP60, -70 and -90. All are ATP-dependent chaperones and require co-chaperones for effectiveness. Low-molecular-weight HSPs are independent of ATP [7,8].

The canine species differs from other carnivores concerning some features of the sexual cycle and pregnancy development and maintenance. The life span of the corpus luteum in pregnant and non-pregnant diestrus dogs is similar; however, luteolysis ante partum requires placental release of prostaglandins, a mechanism initiated by a decrease in PR expression at placentation sites [9]. There is no known factor initiating the recognition of pregnancy. During early pregnancy, the developing canine embryo initiates immunological changes in the intrauterine milieu, finally enabling cell invasion and placenta formation. Later on, the embryo itself and the developing feto-maternal unit are responsible for the maintenance of pregnancy, with implantation and continuous decidualization being determining events [9]. In the beginning, embryo growth and placenta formation, among other things, are possible because of a balanced interplay between proliferation and apoptosis [10,11,12,13,14,15]. During human pregnancy, HSP70 regulates autophagy and apoptosis [16]. In a former study, we detected that in pregnant bitches, serum concentrations of HSP60 and HSP70 were significantly decreased between days 7 and 21 of gestation [17]. We supposed that this might mirror a controlled increase in apoptosis within the uterine compartment during trophoblast cell invasion; this should be proven within the present study, in which we observed development until the postimplantation stage. HSP70 hinders the activation of apoptosis; similar regulatory functions are thought to occur during folliculogenesis and during corpora lutea formation and regression. We therefore mainly aimed to investigate the protein expression of HSP60 and HSP70 in canine uterine and placental tissue by means of immunohistochemistry. Changes in a marker of apoptosis, caspase 3, and an indicator of proliferation, KI67, were investigated in parallel. As tissue collection included the ovaries, and expression of HSP60 and -70 in the canine ovaries is unknown, we additionally examined the expression of these factors within the follicles and corpora lutea. Our hypothesis was that during pregnancy and towards postimplantation, the intrauterine expression of caspase 3 and both regulatory HSPs would change in comparison with that in non-pregnant dogs.

## 2. Materials and Methods

### 2.1. Ethical Approval

This study was approved by the local ethical committee (local ethics committee for animal experiments of Ondokuz Mayıs University, Turkey; approval number 2020/57 (22 October 2020).

### 2.2. Animals

Uterine tissue from 26 healthy female dogs was collected during routine ovariohysterectomy (OHE). The dogs belonged to different breeds (1 King Charles spaniel, 1 Terrier, and 24 mongrels). Selection criteria: All dogs were adult, privately owned and introduced for contraceptive purposes within a project for population control. The dogs were either non-pregnant or mismated and pregnant (postimplantation), and an ovariohysterectomy was required. In dogs close to estrus, some owners allowed for planned mating before the OHE; these animals were mated, ovariohysterectomy was performed after day 10, and then the uterus was flushed for embryos (the dogs were afterwards related to the pregnant or non-pregnant group). No animal had received any prior hormonal treatment. All tissues were obtained with the written consent of the owners.

### 2.3. Methods

All animals were clinically and sonographically examined and healthy. In pregnant animals, vitality of the fetuses and normal appearance of the placentas and fetal fluids was a prerequisite.

Embryo flushing: In dogs mated during estrus and introduced 10–12 days later for embryo flush, the estrus cycle stage before mating was determined by means of clinical findings, vaginal cytology, and progesterone (P4) measurement before OHE. For the latter, an established ELISA was used [18]; the intra- and inter-assay coefficients of variation (%CVs) were calculated as 3.06% and 6.45%, respectively. To assess the linearity and accuracy, serial dilutions of high-concentration serum samples were prepared and compared with expected concentrations. The mean recovery for P4 was within the acceptable range of 92–106%, confirming assay accuracy. The results are expressed in ng/mL.

Mating was completed at 2 days after ovulation when the P4 level was 5–10 ng/mL, at 1 day after ovulation when the P4 level was >10–14 ng/mL, and on the same day when the P4 level was 15–17 ng/mL [19]. Embryo flushing was performed at day 10–12 after mating and after OHE (*n* = 6 positive, *n* = 8 negative). Flushing was performed before tissue collection by using an embryo filter and PBS solution. Considering the stress-responsiveness of heat shock proteins, particular attention was paid to minimizing the pre-fixation handling time. Embryo flushing was performed immediately after ovariohysterectomy, and the time between uterine devascularization and embryo fixation did not exceed 15 min. The tips of the uterine horns and the cervix were closed with a clamp. Then, a needle was inserted into the tip of each horn, and both horns were flushed with PBS while the clamp was removed from the cervix. The fluids were flushed through the filter and the embryos retained. Embryos were placed in a Petri dish and counted, and their developmental stages were determined using a monocular microscope. Dogs from which no embryos were collected were allocated to the early diestrus group.

Examinations of pregnant and diestrus dogs: In all animals introduced for ovariohysterectomy, the cycle stage was determined and pregnancy was excluded/confirmed sonographically. Non-pregnant dogs at day 30–40 of the sexual cycle were included in the late diestrus group (*n* = 6). In pregnant animals, the gestational age was determined sonographically before OHE [20] and anatomically after OHE (pregnant: days 30–40, *n* = 6).

Ovariohysterectomy: A blood analysis was completed before each operation. The operations were performed under general anesthesia in the Kafkas University Faculty of Veterinary Medicine, Dept. of Obstetrics and Gynecology, Kars, Turkey. After the operations, all dogs received NSAIDs for 2 days (Meloxicam, 0.2 mg/kg bw) and were released into home care the same day.

Sampling: Finally, the dogs were assigned to 4 groups: preimplantation days 10–12 (embryo-positive, *n* = 6), postimplantation days 30 to 40 (*n* = 6), early diestrus days 10–12 (embryo-negative, *n* = 8), and late diestrus days 30–40 (*n* = 6). All tissues from non-pregnant animals served as controls. After each operation, one ovary (with follicles/corpora lutea) was taken, and one tissue sample of size 2 × 2 cm was taken from the middle of the uterine horn (pregnant animals: interplacental site); in postimplantation dogs (day 30–40; *n* = 6), placental tissue (2 × 2 cm) was also collected (see Table 1).

Samples were placed in 4% buffered formalin for 24 h and then transferred into 70% ethanol (ETOH) for a maximum of 14 days. All ovaries and uteri were examined anatomically and histologically for pathologies; only physiological samples were analyzed.

**Figure 1:**
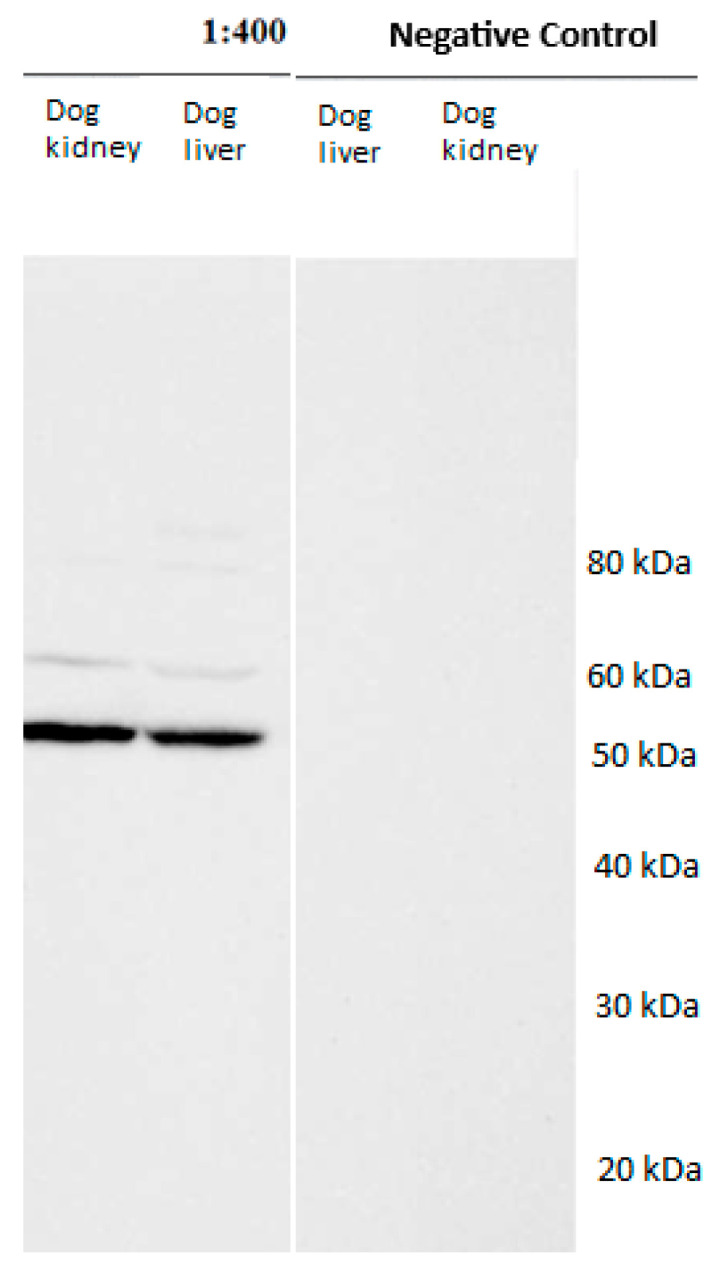
Western blot analysis with the HSP60 primary antibody (ABIN1498529) using homogenized lysates from canine liver and kidney. The negative control sections were processed as described, except that they were incubated with PBS instead of the primary antibody. Note the marked bands at 60 kDa.

**Figure 2:**
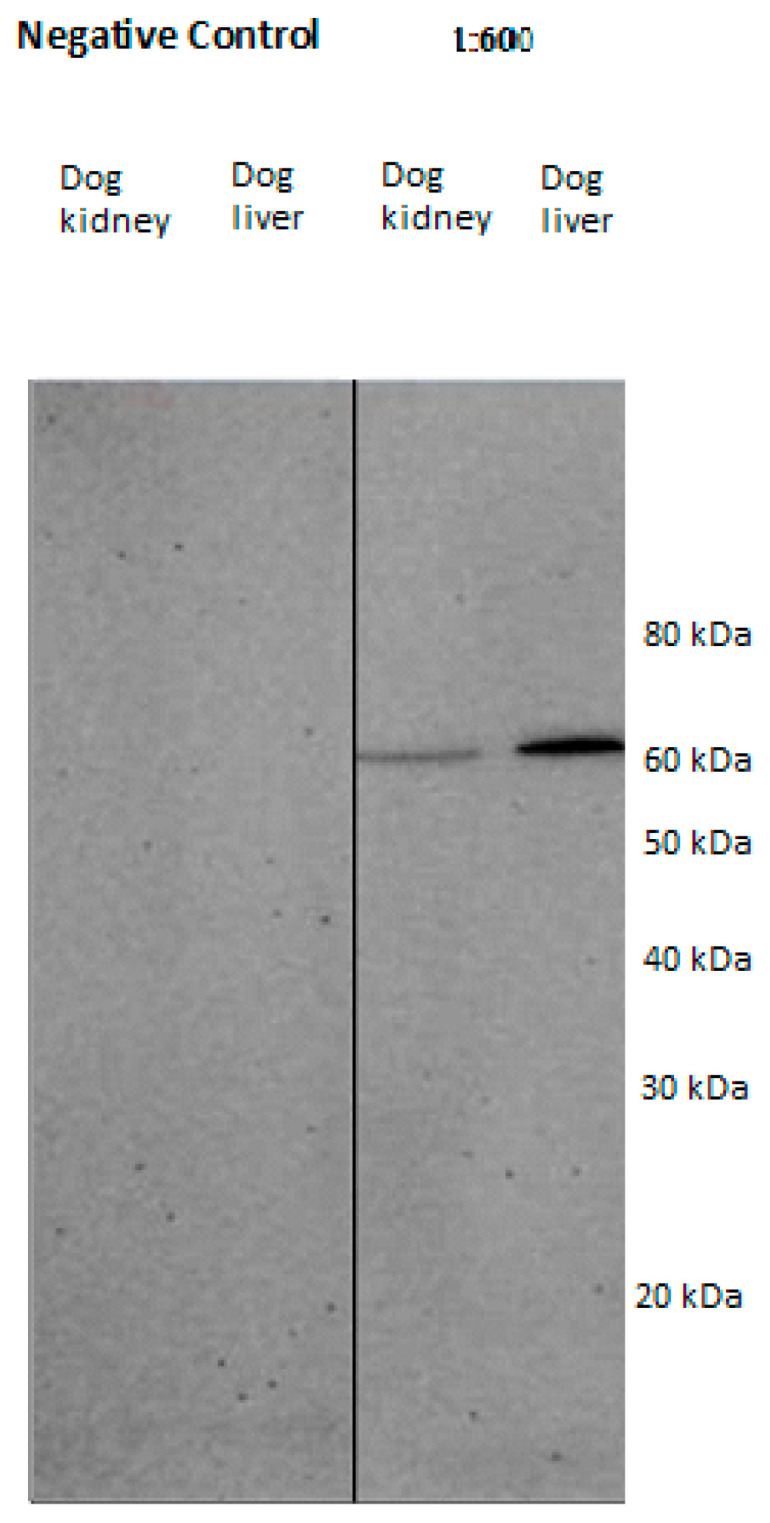
Western blot analysis with the monoclonal HSP70 primary antibody (ABIN361707) using homogenized lysates from canine liver and kidney. The negative control sections were processed as described, except that they were incubated with PBS instead of the primary antibody. Note the marked bands at 70 kDa.

Western blot: The primary antibodies against HSP60 and -70 used for immunohistochemistry were tested for their specificity by means of Western blot. Western blot analyses were performed at the Institute of Morphology, University of Veterinary Medicine Vienna (A), as described by [21]. Liver and kidney tissues from dogs were shock frozen in liquid N_2_ and minced. Protein lysates were produced in RIPA lysis buffer (50 mM Tris-HCl pH 7.4, 500 mM NaCl, 0.5% natriumdeoxycholat (Carl Roth GmbH, Karlsruhe, Germany), 1% Nonidet P-40 (Igepal, Sigma Aldrich, Wien, Austria), 0.1% natriumdodecylsulfat (Serva, Heidelberg, Germany) and SDS lysis buffer (62.5 mM Tris-HCl, pH 6.8, 50 mM DTT (Carl Roth GmbH, Vienna, Austria), 2% SDS (Merck, Vienna, Austria), 10% glycerol (Serva, Heidelberg, Germany), and after the addition of 1% (*v*/*v*) protease and phosphate inhibitor (Protease-Inhibitor Cocktail and Phosphatase-Inhibitor Cocktail 3, both Sigma Aldrich, Vienna, Austria) using a Dounce homogenizer. Lysates were incubated for 30 min on ice, vortexed, repeatedly pulled through a 20 g needle and finally centrifuged at 10,000 rpm for 15 min. The protein concentration was measured by using a DC™ Protein Assay (Bio-Rad, Vienna, Austria) according to manufacturer’s instructions. Protein extracts were placed on a 10% polyacrylamide minigel and then transferred onto PVDF membranes (GE Healthcare, Tiefenbach, Austria). To prevent unspecific binding, the membranes were blocked using Western blocking reagent (Roche, Vienna, Austria) 1:10 in TBST for 2 h at RT. Incubation with the primary antibody (Table 1) was performed at 4 °C overnight. All antibodies were diluted in Western blocking reagent (Roche, Vienna, Austria)/TBST (1:10). The negative control sections were processed as described, except that the negative control sections were incubated with PBS instead of the primary antibody. Signals were made visible with the Amersham Western Blotting Detection Reagent (GE Healthcare, Tiefenbach, Austria) and the BioRad ChemiDoc Image System using Image Lab Software 6.1 (Bio-Rad, Vienna, Austria). Both antibodies produced positive bands with the control tissues (Figure 1 and Figure 2) and no bands without the antibodies (negative control).

Immunohistochemistry: After 24 h of sample storage in formalin, formalin was exchanged with 70% ethanol. Embedding in paraffin was completed within the following 14 days. Paraffin blocks were serially sectioned at 2 µm and placed on glass slides. Samples were prepared for immunohistochemistry staining following the routine procedure at the Institute for Morphology, University of Veterinary Medicine Vienna (A). Briefly, samples were de-paraffinized with xylene (2 × 8 min), 100% ethanol (2 × 3 min), 96% ethanol (1 × 3 min) and 70% ethanol (1 × 3 min). Endogen peroxidases were blocked in 40 mL methanol + 10 mL 3% H_2_O_2_ + 90 mL Aqua dest. for 15 min at room temperature. After 10 flushes with water, antigen demasking was performed with the Heat Induced Epitope Retrieval (HIER) technique, with samples placed in Tris-EDTA (pH9) in a steamer at 85 °C for 30 min. Samples were then flushed for 2 × 5 min in phosphate-buffered saline (PBS; pH 7.4), covered with normal goat serum 1.5% and stored for 30 min in a wet chamber at room temperature. The primary antibody (Table 1) was diluted with PBS, and the slides were incubated at 4 °C overnight (negative controls: without antibody; positive controls: canine lymph nodes). The following day, the slides were washed with PBS (2 × 5 min); then, the samples were incubated with the diluted secondary antibody (Table 2) for 30 min in a wet chamber.

Afterwards, the slides were washed with PBS and incubated with the chromogen DAB for 5 min in the wet chamber at room temperature, followed by washing with aqua dest. for 10 min. Counterstaining was performed with Mayer’s hematoxylin for 3 min, then the slides were again washed with aqua dest. for 10 min. Finally, the samples were dehydrated in ethanol (96% 1 × 3 min, 100% 2 × 3 min, xylol 2 × 3 min). The samples were automatically covered with a cover glass using Dibutylphthalat Polystyrol Xylol (DPX) mountant (Merck Millipore, Vienna, Austria). Two slides from each group were stained with hematoxylin and eosin (H&E). The negative control sections were processed as described, except that they were incubated with PBS instead of the primary antibody. Positive controls were prepared using canine lymph node tissue.

Microscopic evaluation: Stained samples were evaluated using a Zeiss Axiolmager Z2 microscope equipped with digital camera (Carl Zeiss Microscopy GmbH, Jena, Germany) and photographed using the software ZEN Version 3.10 (Carl Zeiss Microscopy GmbH, Jena, Germany). Eight areas per section were randomly selected and evaluated. The pictures were evaluated quantitatively (nuclear staining, HSP70 and Ki67) and qualitatively (prevalence and intensity of the immunosignal, cytoplasmic staining, HSP60 and caspase 3). Evaluations were completed by two independent researchers. For quantitative assessment of the stained nuclei, the open-source platform FIJI was used, but the cells were counted manually (open-source software ImageJ 1.x) and the number of stained nuclei per group was used for statistical calculations. For the qualitative evaluation, the amount of staining (0%, 0–25%, 25–50%, 50–75% or 75–100% [24,25]) was estimated per slide (negative, weak, moderate or strong [26]); the intensity was scored subjectively and merely described. Only high-quality pictures were evaluated, and the edge of the tissues was avoided. Batch effects can be excluded as the fixation and staining procedures and the scanning and imaging techniques were consistent.

### 2.4. Statistical Analyses

The sample size was calculated using *G Power 3.1.9.2. For all tests, IBM SPSS statistics version 24 (SPSS Ltd., Hong Kong, China) was used. Comparisons of data with a normal distribution were performed using the Kolmogorov–Smirnov test. As most data were not normally distributed, comparisons within and between groups were performed using the Friedman test and the Mann–Whitney U test. Statistical significance was set at *p* < 0.05. All results are given as the average ± standard deviation (SD).

## 3. Results

### 3.1. Pregnancy

**Figure 3:**
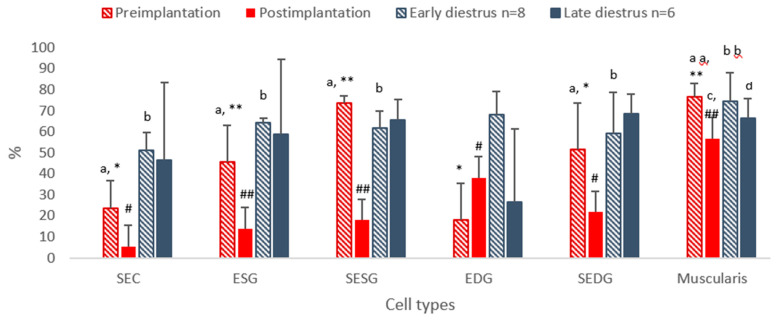
Protein expression of HSP70 in uterine tissue of pregnant and non-pregnant dogs. Percentage of positive cells per group. SEC, superficial epithelial cell; ESG, endometrial superficial gland; SESG, stroma of endometrial superficial gland; EDG, endometrial deep gland; SEDG, stroma of endometrial deep gland; muscularis, smooth muscle layer of uterine wall. Preimplantation = preimplantation phase of gestation (days 10–12 after ovulation, *n* = 8); postimplantation—postimplantation phase of gestation (days 30–40, *n* = 6); early diestrus—non-pregnant uterus at days 10–12 (*n* = 8); late diestrus—non-pregnant uterus at days 30–40 (*n* = 6); a:b *p* < 0.05, aa:bb *p* < 0.01, *:# *p* < 0.05, **:## *p* < 0.05, c:d *p* < 0.05.

In the preimplantation phase of early gestation, HSP70 was ubiquitously expressed; positive cells predominated in the stroma of endometrial superficial glands (SESGs 73.4%) and endometrial deep glands (SEDGs 51.4%), and in the muscular layer (76.4%). Furthermore, endothelial cells of blood vessels were weakly stained. All positive cell signals significantly decreased towards postimplantation (*p* < 0.05–*p* < 0.01; Figure 3).

Most cells in all layers stained positive for HSP60, though the staining was in some dogs lesser and only in stroma cells; the staining intensity was especially high in the superficial compartment (superficial endometrial cells (SECs), endometrial superficial glands (ESGs), endometrial superficial glands (SESGs); 2.7) but also mostly high grade in the endometrial deep glands (EDGs; Figure 6).

**Figure 4:**
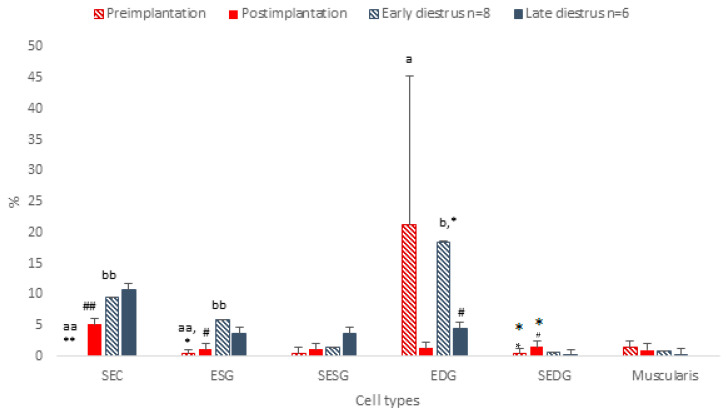
Protein expression of Ki67 in uterine tissue of pregnant and non-pregnant dogs. Percentage of positive cells per group. SEC, superficial epithelial cell; ESG, endometrial superficial gland; SESG, stroma of endometrial superficial gland; EDG, endometrial deep gland; SEDG, stroma of endometrial deep glands; muscularis, smooth muscle layer of uterine wall. Preimplantation = preimplantation phase of gestation (days 10–12 after ovulation, *n* = 8); postimplantation—days 30–40 of gestation (*n* = 6); early diestrus—non-pregnant uterus at days 10–12 (*n* = 8); late diestrus—non-pregnant uterus at days 30–40 (*n* = 6); a:b *p* < 0.05, aa:bb *p* < 0.01, *:# *p* < 0.05, **:## *p* < 0.01.

**Figure 5:**
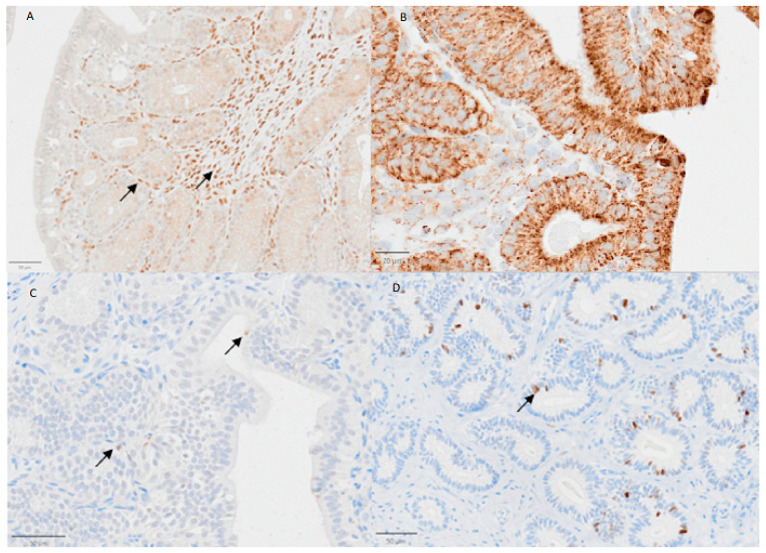
Protein expression of HSP60, HSP70, Ki67 and caspase 3 in the uterus of a female dog during the preimplantation stage. (**A**) Arrows: Positive, brown signals for HSP70 in stroma and endometrial glands. (**B**) Arrows: Positive, brown signals for HSP60 in cytoplasm of SECs and glands. (**C**) Arrows: Weak positive, brown signals for caspase 3 in SECs and gland. (**D**) Arrow: A positive, brown signal for Ki67 in the DEG. The bar indicates 50 µm (B: 20 µm).

Few Ki67-positive cells were detected. In the EDGs, the expression level reached 21.3%, but in all other cells, the percentage varied between 0.4 and 1.4%. The expression of Ki67 in the SECs and ESGs was significantly lower than that in non-pregnant animals (*p* < 0.01; Figure 4 and Figure 5).

Most cells were caspase 3-negative; weakly stained cells were mainly seen in the superficial compartment (SECs, ESGs and SESGs; Figure 5).

In uterine samples taken between days 30 and 40 of pregnancy, the number of HSP70-positive cells was significantly decreased in comparison with the preimplantation stage (*p* < 0.05–*p* < 0.01; Figure 3); in the muscular layer, positive cells predominated (>56.7%), while expression in all other cell types was low, especially in the SECs. Expression was mostly significantly lower than that in non-pregnant animals, except in the EDG (*p* < 0.05; Figure 3).

During the postimplantation stage, in most cells, there was a decrease in the HSP60 staining intensity but not in the number of HSP60-positive cells. The intensity of staining was highly variable among the dogs and mostly lower than that in the preimplantation stage.

The percentage of Ki67-positive cells was highest in the SECs and significantly increased in comparison with that in the preimplantation stage (5.2%, *p* < 0.05) (Figure 4). There was no significant difference between the postimplantation stage and late diestrus.

The number of stained cells and staining intensity of caspase 3 were low in all layers and did not change towards postimplantation; the muscular layer was negative in all dogs.

**Figure 6:**
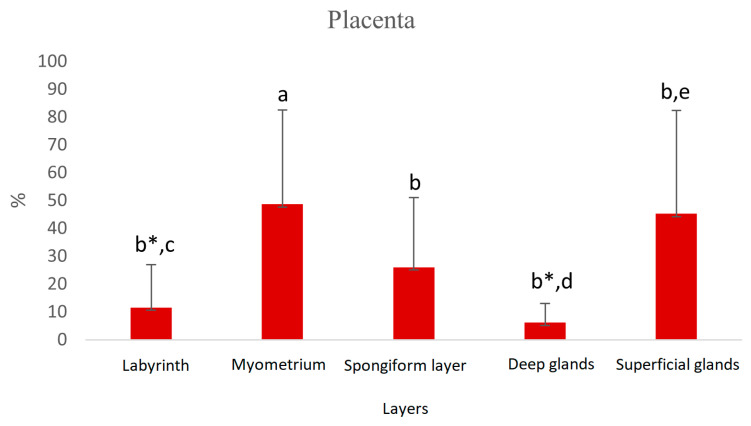
Protein expression of HSP70 in placental tissue of pregnant dogs. Percentage of positive cells. Labyrinth—placental labyrinth; myometrium—smooth muscle layer of uterine wall; a:b *p* < 0.05, a:b* *p* < 0.01, c:d *p* < 0.05, c:e *p* < 0.01.

**Figure 7:**
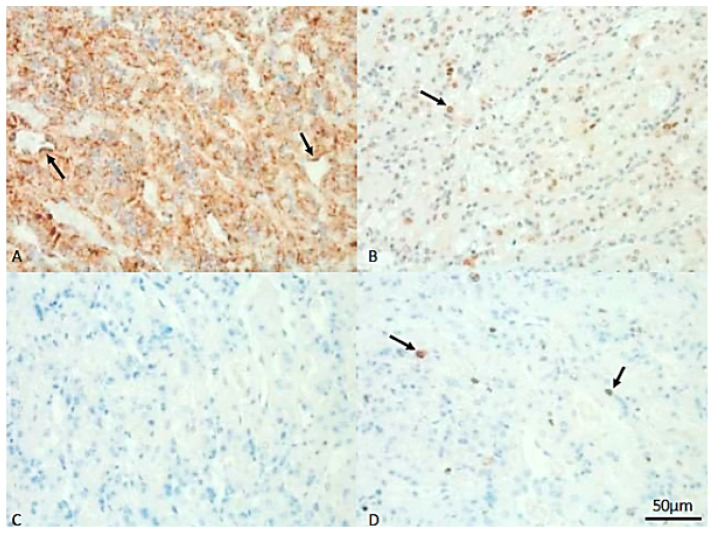
Protein expression of HSP60, HSP70, Ki67 and caspase 3 in the placental labyrinth of a female dog. (**A**) Arrows indicate positive, brown signals for HSP60 in endothelial cells of blood vessels. (**B**) The arrow indicates positive (brown) staining for HSP70 in the nucleus of a cytotrophoblast cell. (**C**) Unstained cytotrophoblast tissue after incubation with caspase 3-specific antibodies. (**D**) Expression of Ki67 in nuclei of cytotrophoblast cells (arrows). The bar indicates 50 µm for all sections.

**Figure 8:**
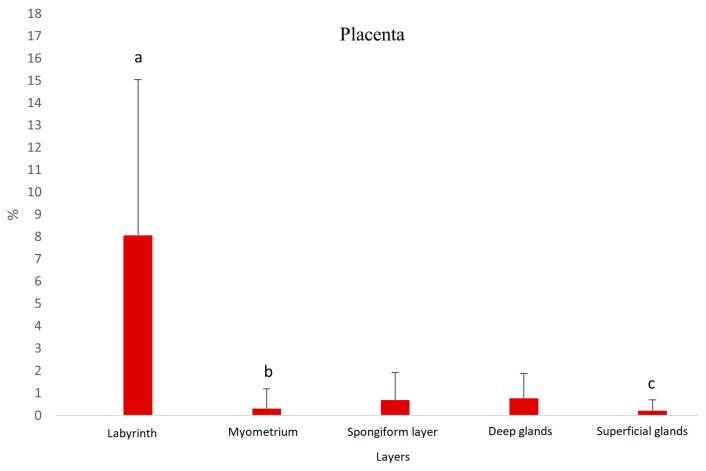
Protein expression of Ki67 in placental tissue of pregnant dogs. Percentage of positive cells. Labyrinth—placental labyrinth; myometrium—smooth muscle layer of uterine wall; a:b *p* < 0.01, a:c *p* < 0.05.

In the placenta, HSP70 was mainly expressed in the nuclei and cytoplasm of the muscular layer, in superficial glands and in myocytes surrounding the blood vessels. Maternal endometrial stromal cells and decidual cells stained positively for HSP70, whereas endometrial epithelial cells lacked an immunosignal for HSP70 (Figure 5, Figure 6 and Figure 7B). In fetal regions, syncytiotrophoblasts gave a positive HSP70 nuclear signal (Figure 8). The endothelial cells of blood vessels were weakly stained.

A cytoplasmic signal for HSP60 was ubiquitously detected in fetal and maternal tissue in granular patterns and with variable intensity; the HSP60 signal was mostly visible in the trophoblast, in both layers of the smooth muscle cells and in the endothelial cells of blood vessels (Figure 7A).

The Ki67 immunosignal was mainly detected but with high variability in cells of the placenta labyrinth; vascular endothelial cells were negative (Figure 8). Staining in the syncytiotrophoblast was stronger than that in the cytotrophoblasts (Figure 7D). The caspase 3 signal was weak in most samples and cell types and negative in the placenta labyrinth (Figure 7C).

**Figure 9:**
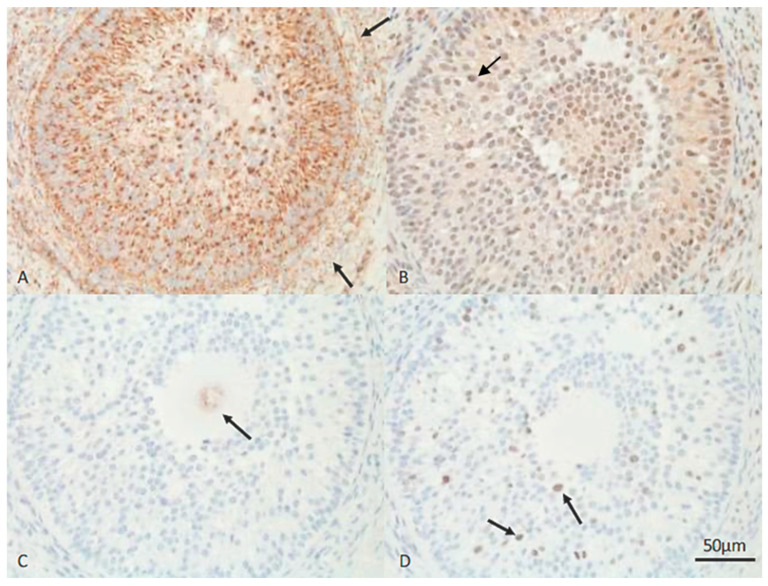
Protein expression of HSP60, HSP70, Ki67 and caspase 3 in the follicle of a pregnant female dog. (**A**) Follicular tissue; the arrows indicate theca interna cells with a positive HSP60 signal in the cytoplasm. (**B**) Follicular tissue; the arrow indicates a positive HSP70 nuclear signal. (**C**) No signal after staining with caspase 3; the arrow indicates the ovum. (**D**) Strong nuclear staining for Ki67 in granulosa cells of a follicle. The bar indicates 50 µm for all sections.

**Figure 10:**
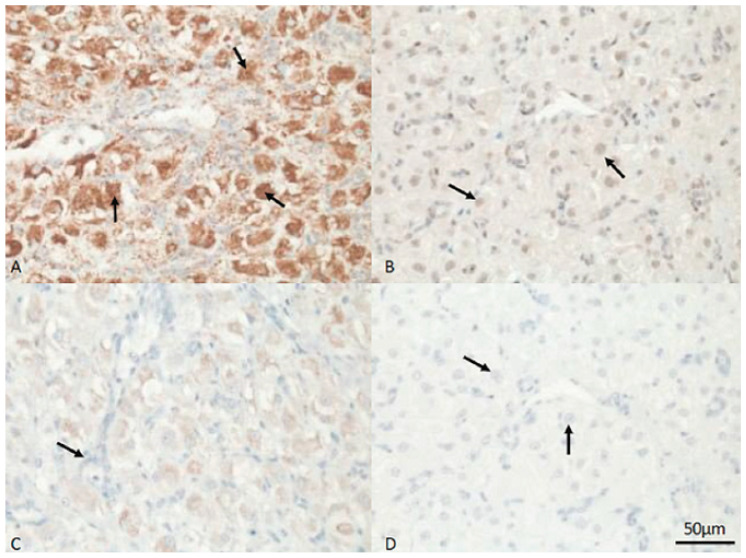
Protein expression of HSP60, HSP70, Ki67 and caspase 3 in the corpus luteum of a pregnant female dog. (**A**) Corpus luteum tissue; the arrows indicate granulosa lutein cells with positive HSP60 signal in the cytoplasm. (**B**) Corpus luteum tissue; arrows indicate positive HSP70 signals in the nuclei of granulosa lutein cells. (**C**) No signal after staining with caspase 3. (**D**) No signal after staining with Ki67 in granulosa lutein cells. The bar indicates 50 µm for all sections.

In all developmental stages of follicles, the expression of HSP 70 was extremely high, without any significant difference among the examined cells and pregnancy stages. The same applies to HSP60; however, in most samples, the number of positive cells and intensity of staining increased towards postimplantation. The number of positive signals for caspase 3 was low; however, granulosa cells of tertiary follicles and oocytes more frequently stained positive, and the staining intensity increased towards postimplantation. The expression of KI67 in follicles was very low until postimplantation; however, the number of positive signals increased with the developmental stage, and such signals were mainly observed in inner granulosa cells (Figure 9 and Figure 10).

In the corpora lutea, there was a striking increase in the expression of HSP70 from early pregnancy towards postimplantation. Similarly, in all cells of the corpora lutea, the expression of HSP60 was 75–100%, reaching 100% during postimplantation; the intensity of staining increased as well. Staining was especially strong in the external theca lutein cells (Figure 10). In addition, blood vessel endothelial cells and connective tissue stained positive. The staining intensity for caspase 3 was highly variable and strongest in the theca lutein cells of the corpora lutea. There was no expression of Ki67 in the corpora lutea during pre- or postimplantation (Figure 10).

### 3.2. Diestrus

During early diestrus, the expression profile of HSP70 resembled that in early pregnancy, but with significantly higher expression in the SECs, ESG, SEDG (all *p* < 0.05) and EDG in comparison with pregnant animals. No significant change in cell signals was seen between early and late diestrus for any cell type (Figure 3).

Concerning HSP60, fewer cells than in the preimplantation stage were positively stained; similarly to those in the preimplantation stage, few stroma cells were stained, as were few muscular layer cells. However, the staining intensity appeared comparably high, especially in the SECs and gland cells.

For KI67, the scheme resembled that in the preimplantation stages, but with significantly higher expression in the SECs (9.53%, *p* < 0.01) and ESG (6%, *p* < 0.01) than in pregnant animals (Figure 4). Mostly, no change was observed towards late diestrus, except a significant decrease in the positive signal in the EDG (*p* < 0.05).

During early and late diestrus, few cells stained caspase 3-positive, and the intensity score was mostly variable and weak as in the preimplantation dogs; only in the stroma and the DEG did the staining intensity approach medium grade in some dogs. Most cells were negative.

**Figure 11:**
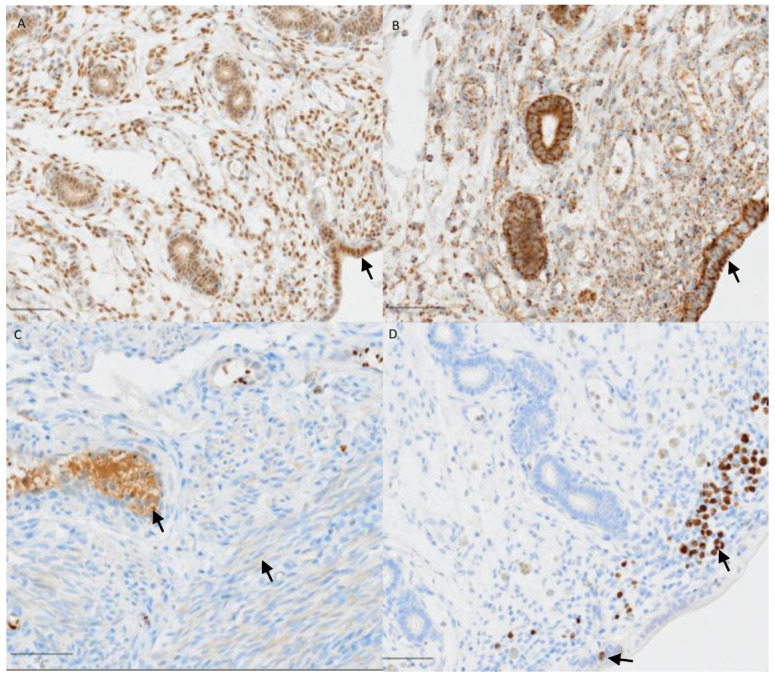
Protein expression of HSP60, HSP70, Ki67 and caspase 3 in the late diestrus uterus of a female dog. (**A**) Arrow: positive, brown signals for HSP70 in SECs. (**B**) Arrow: positive (brown) staining for HSP60 in SECs. (**C**) Arrows: positive, brown signals for caspase 3 in the muscularis and glands. (**D**) Arrows: positive, brown signals for Ki67 in SECs, glands and stroma. The bar indicates 50 µm for all sections.

Between days 30 and 40 of diestrus, HSP70-positive cells still predominated except in the endometrial deep glands (EDGs), where HSP70 expression was significantly decreased (from 68.2 to 26.7%, *p* < 0.05). On day 30, more cells were positive in non-pregnant animals than in pregnant dogs, except in the EDGs (Figure 3 and Figure 11).

In all dogs and cell types, the number of HSP60-positive cells increased until late diestrus to 76–100%, including the muscular layer. The staining intensity was highly variable (Figure 10).

On day 30 of diestrus, Ki67 expression was generally similar to that at mid-gestation in all cell types except the SEDG and muscular layer; however, the difference was non-significant (Figure 4 and Figure 11). In the EDG, significantly lower numbers of cells stained positively in the postimplantation and late diestrus stages in comparison with preimplantation and early diestrus (*p* < 0.05, Figure 4 and Figure 11).

The percentage of caspase 3-positive cells increased to 75% in single dogs, especially in the ESG, SESG and muscular layer; however, most cells were still negative. The staining intensity was highly variable but increased in some dogs, even to medium or high grade, especially in the superficial compartment (SECs, ESG, SESG; Figure 11).

In the corpora lutea and follicles of diestrus dogs, HSP70 expression resembled that in pregnant animals. Positive signals for HSP60 in the corpora lutea and follicles were highly variable (25–100%), as was the intensity of staining. KI67 expression in the corpora lutea and follicles did not differ from that in pregnant animals, and caspase 3 was barely expressed in the corpora lutea and follicles of non-pregnant dogs.

## 4. Discussion

The aim of this study was to compare the expression of HSP60 and HSP70 between pregnant and non-pregnant dogs, since these chaperones are known to regulate autophagy and apoptosis and to contribute to cell shelter and repair during pregnancy in other species such as humans and mice [3,16]. During a pilot study, we were able to show that these chaperone proteins are differentially expressed in the canine uterus and placenta and are detectable in the canine syncytiotrophoblast and cytotrophoblast [27]. Only recently, we found protein expression of both chaperones in the preimplantation embryo [28]. In this study, we went into depth and directly compared the findings in pregnant animals with those in non-pregnant tissues (from corresponding cycle stages) and found some striking differences. During the preimplantation stage, the feto-maternal interaction changes the intrauterine milieu, with changes in the endometrium and uterine wall being the first to be detected. During early gestation, HSP70 was strongly and ubiquitously expressed. Its expression in superficial cells was significantly decreased in comparison with that in non-pregnant animals. Similarly, in humans, HSP70 is already detectable in uterine tissue during the preimplantation stage of gestation [22], and in pregnant gilts, it has been shown to be upregulated until day 12 post insemination [29]. Interestingly, in the latter species, HSP60 is down-regulated during this period [29]. In this study, the significant decrease in protein expression of the anti-apoptotic agent HSP70 in the superficial part of the endometrium in the postimplantation stage was striking. It may indicate a change, as intense apoptosis of the maternal tissue during implantation requires regulation, which may be less important after decidualization; however, this is hypothetical. Interestingly, expression of caspase 3 was mainly detected in cells belonging to the superficial compartment. The balanced regulation of proliferation and apoptosis determines the success of pregnancy establishment and development. During a former study, we investigated the mRNA expression of FAS and FAS-L during canine pregnancy between days 10 and 45 of gestation. These factors, activated by extrinsic factors such as TNF ligands during inflammation, are known to regulate apoptosis during implantation in humans and other species; an increased rate of apoptosis is important for successful establishment of pregnancy [30,31]. We found a significant decrease in FAS-L during implantation and thereafter, while the expression of FAS remained almost unchanged [12]. As apoptosis may have extrinsic and intrinsic causes [32,33], in this study, the protein expression of caspase 3, induced by DNA damage, was examined, and we found the immunosignal in the superficial compartment to be rather intense. A gradual increase in caspase 3 immunoreactivity was similarly found in the pregnant rat uterus during implantation and progressing gestation [34]. Furthermore, during this early stage of gestation, few KI67-positive cells were detected; this makes sense, as the early stage is mainly hallmarked by maternal tissue apoptosis. On the other hand, HSP60-positive cells were most frequently detected in the superficial compartment and later in the placenta, but also in the corpora lutea and follicles. HSP60 completes multiple important tasks in different body cells. In Madin–Darby canine kidney renal tubular cells, knockdown of HSP60 using small interfering RNA (siRNA) increases the amount of intracellular protein aggregates and unfolded proteins, oxidative stress and ATP production, which can lead to cell death [1,35]. Strong immunostaining for HSP60 is detectable in male dogs with prostate gland carcinoma [36] and in dogs with osteosarcoma, especially in dogs with a shorter survival time [37]; these conditions are attended by strong tissue lesions and dedifferentiation. In the preimplantation uterine stromal cells of pregnant mice, treatment with LPS induced a severe decrease in HSP60 expression and an increase in DNA damage as compared with non-pregnant animals [38]. These facts might indicate a protective effect of HSP60, probably also during phases of large reorganization of the feto-placental compartment such as during early gestation. We had expected downregulation in parallel to that of HSP70; however, the functions of HSP60 and -70 are probably different and must be further investigated. The strong cytoplasmic immunosignal during early gestation in all these tissues may explain why the serum concentration was significantly decreased between days 7 and 21 in comparison with that in non-pregnant female dogs in a former study [17]; however, this cannot be proven at present.

In the uterus, the intensity but not the number of positive signals of cytoplasmic HSP60 staining decreased during the postimplantation stage, when decidualization was complete. In comparison, HSP60 expression in non-pregnant tissues was weaker and more variable, including in the corpora lutea and follicles. Implantation is the phase of intense stroma modification and decidualization, which requires trophoblast growth and other mechanisms such as PGE2 and progesterone (P4) initializing mesenchymal–epithelial decidual transition, and, to a lesser degree, the HSPs [39,40]; with increasing decidualization, apoptotic mechanisms and anti-apoptotic factors lose their relevance [41]. We hypothesize that changes were therefore detected in the postimplantation phase, after placenta formation. Proliferation was ongoing, as KI67 expression was significantly increased especially in the SECs, while caspase 3 expression did not change and HSP70 expression was significantly decreased, pointing towards the maternal tissue reacting to invasive embryo growth by allowing for apoptosis. Different expression in pregnant animals was one hypothesis and could be proven within this study. The high expression of KI67 in the placenta labyrinth and of anti-apoptotic HSP70 in the trophoblasts appears logical as the placenta grows quickly during the postimplantation phase.

The expression of HSP70 in the follicles and corpora lutea was higher and more consistent than the immunosignal for HSP60 in pregnant and non-pregnant animals. Members of the HSP70 family, together with HSP40 and HSP90, are co-expressed and up-regulated in mouse ovaries under heat stress [42]. In rats [43], HSP70 was not detectable in the follicles, only in the developing corpora lutea, while it decreased in old corpora lutea and during pregnancy. In contrast, HSP60 was detectable during early stages of folliculogenesis, and its expression increased especially in the granulosa and theca cells. In corpora lutea, the expression of HSP60 did not change during development or regression. In this study, the HSP60 immunosignal was especially strong in the theca cells of the corpora lutea, as was caspase 3 expression, while the intensity of staining in the interstitial and granulosa cells was weaker. In rat interstitial cells, the immunosignal for HSP was stronger during the first days of pregnancy but then decreased until the beginning of lactation. Interstitial cells participate in the synthesis of steroid hormones; furthermore, they regulate folliculogenesis and atresia and provide mechanical shelter [44]. Authors have suggested that in rats, HSP60, together with HSP90, participates in folliculogenesis, luteal development and steroidogenesis in luteal cells, while HSP60 and -70, together with HSP27 and HSP90, participate in luteal regression and steroidogenesis in interstitial cells [43]. In dogs, considering the main tasks of both HSPs and that in this study, the ovaries were not exposed to heat stress, the regulation of apoptosis during folliculogenesis, as well as corporal lutea formation and lysis and steroidogenesis, should be considered and further investigated. While there are differences among species regarding the intensity and expression of HSPs, these might be related to differences in cycle and pregnancy duration and regulation, as well as local and timely differences in the regulation of folliculogenesis and corpora lutea formation/function.

### Study Limitations

The data presented herein were obtained from a relatively low number of patients and must be interpreted with caution. Furthermore, we mainly described protein expression without functional assays. All supposed functions are therefore hypothetical and require further investigations.

## 5. Conclusions

This study provides a basis concerning the distribution of heat shock proteins (HSPs) in the pregnant and non-pregnant canine uterus and ovaries, revealing significant differences. Significant downregulation of HSP70 protein expression in uterine superficial cells during postimplantation is one feature of pregnancy and might mirror the previous need for regulatory anti-apoptotic mechanisms during implantation—mechanisms that are no longer needed after decidualization. The expression of HSP60 did not parallel that of HSP70, pointing towards different functions. The low-grade co-expression and slowly increasing expression of caspase 3 is thought to be a physiological, regulatory factor. In future studies, the additional presence and function of small HSPs would be of interest. Heat shock proteins may provide shelter for undisturbed pregnancy development; however, this requires further investigation.

## Figures and Tables

**Table 1:** Number of samples per group.

Number of Samples (*n*=)/Stage	Ovaries	Placentas	Interplacental Tissue	Uterine Horn
Preimplantation	6			6
Postimplantation	6	6	6	
Early diestrus (non-pregnant)	8			8
Late diestrus (non-pregnant)	6			6
Total (*n*=)	26	6	6	20

**Table 2:** Primary and secondary antibodies for IHC and Western blot.

**Western Blot**					
Primary Antibody	Clonality	Host	Dilution	Company	Code
HSP60	Polyclonal	Mouse	1/400 and 1/500	Antibodies-online.com	Clone 3A2, ABIN1498529
HSP70	Monoclonal	Mouse	1/600	Antibodies-online.com	Clone C92F3A-5, ABIN361707
Secondary Antibody			Dilution	Source	
Mouse IgG HRP-Linked Whole Ab (from Sheep)		Mouse	1/5000	Amersham, England	NA931
**IHC**					
Primary Antibody			Dilution	Source	
HSP60	Polyclonal	Mouse	1/1000	Antibodies-online.com	Clone 3A2, ABIN1498529
HSP70	Monoclonal	Mouse	1/100	Antibodies-online.com	Clone C92F3A-5, ABIN361707
Caspase 3 [22]	Monoclonal	Rabbit	1/250	Cell Signaling, Denvers, USA	Clone 5A1E, # 9664
Ki67 [23]	Monoclonal	Mouse	1/1000	Agilent Technologies,	Clone MIB1, # M7240
Secondary Antibody			Dilution	Source	
For HSP60, HSP70 and KI67 BrightVision Poly-HRP-anti-Mouse		Mouse	Ready to use	Immunologic, Duiven, The Netherlands	DPVM110HRP
For Caspase 3 BrightVision Poly-HRP-anti-rabbit		Rabbit	Ready to use	Immunologic, Duiven, The Netherlands	DPVM110HRP

## Data Availability

The original contributions presented in this study are included in this article/Supplementary Materials. Further inquiries can be directed to the corresponding author.

## Supplementary Materials

The following supporting information can be downloaded at: https://www.mdpi.com/article/10.3390/vetsci13050482/s1, Figure S1: Original image of Figure 1; Figure S2: Original image of Figure 2.
