# Supplementary material for: Heat Shock Proteins 60 and 70, Ki67 and Caspase 3 Are Differentially Expressed in the Canine Pregnant and Non-Pregnant Uterus and Ovaries
Source: Vet Sci. 2026 May 16;13(5):482. doi: 10.3390/vetsci13050482 (PMC13211642; doi:10.3390/vetsci13050482)

vetsci-4275226
Heat Shock Proteins 60 and 70, Ki67 and Caspase 3 are Differentially Expressed in the Canine Pregnant and Non-Pregnant Uterus and Ovaries

Western Blot Data

Densitometry readings/intensity ratio of each band: the western blot was only done to verify specifity of the used antibodies. Densitometry readings/intensity ratio calculations would therefore not make sense

-

Figure S1: Original image of Figure 1

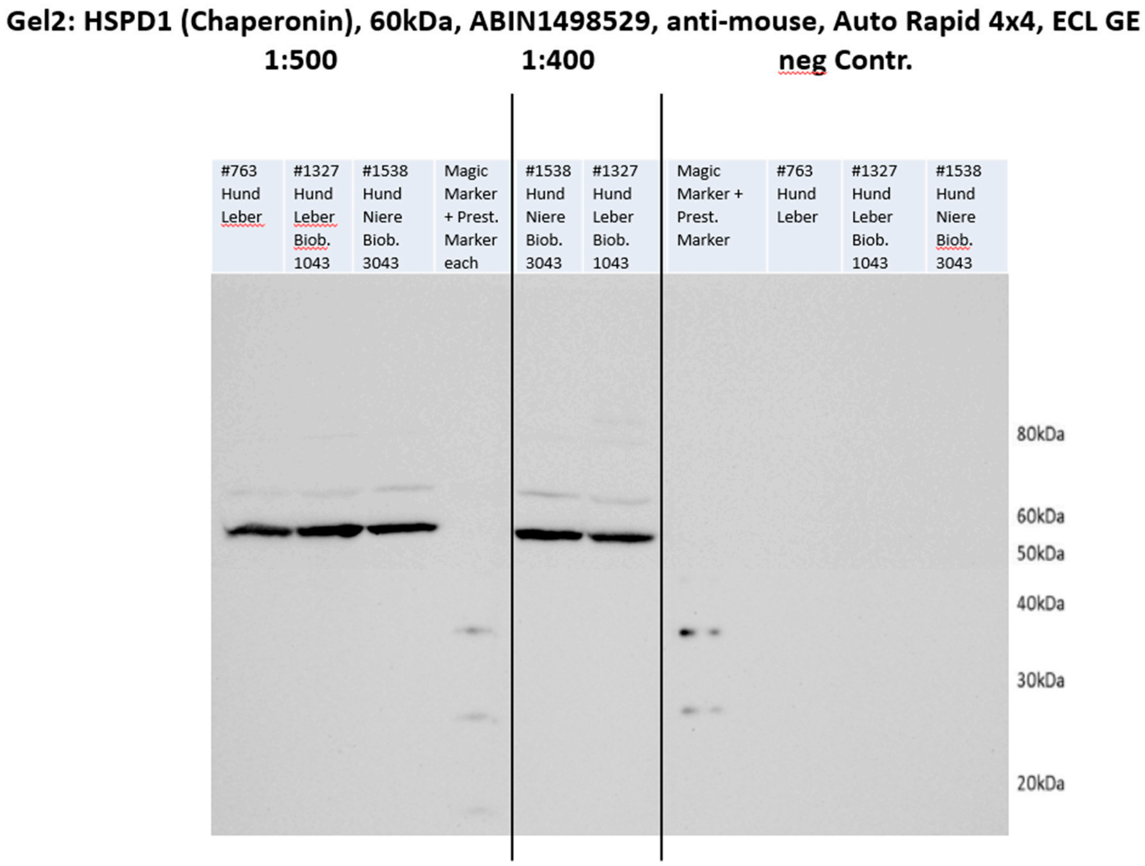

Figure S2: Original image of Figure 2

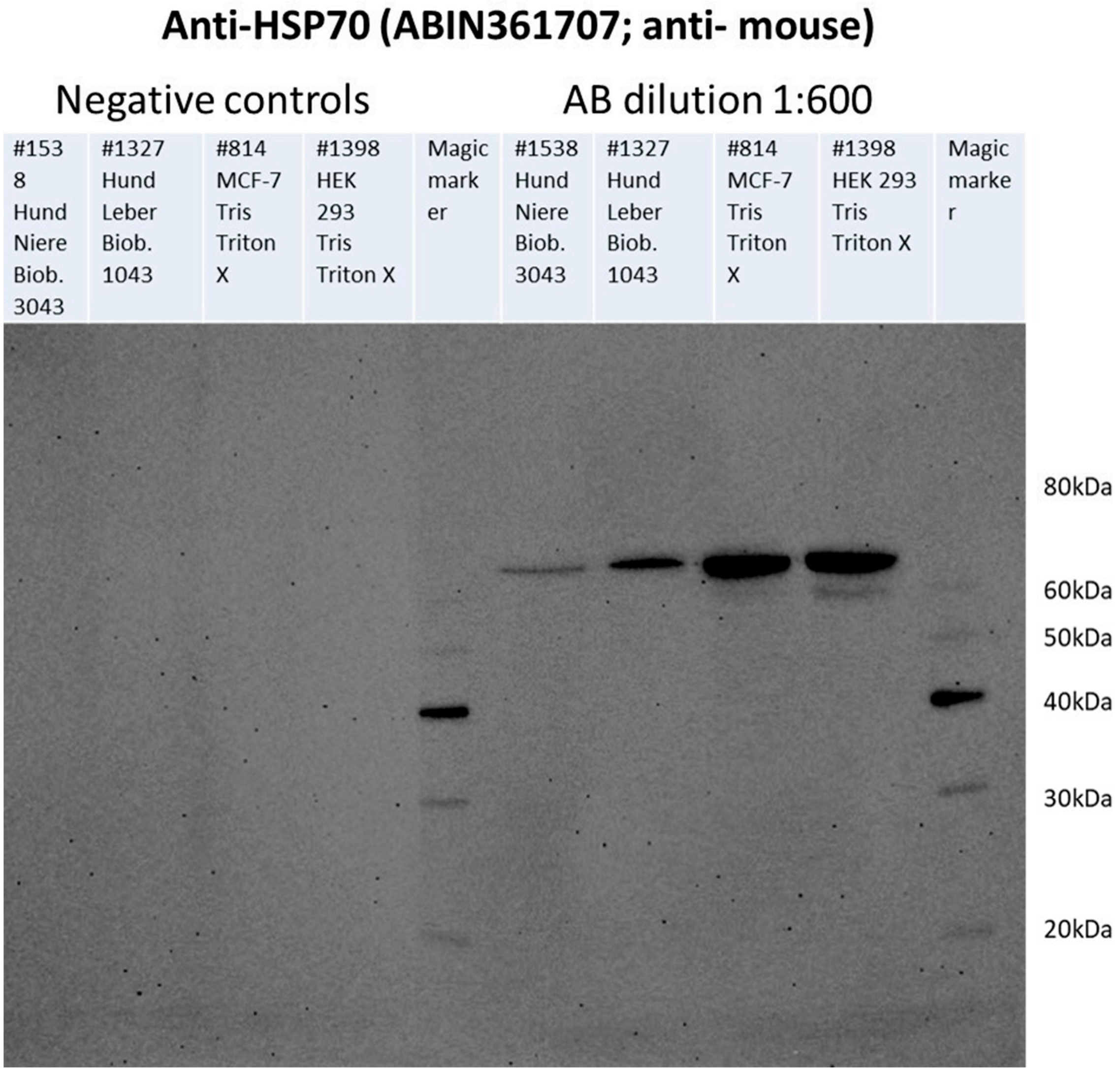

Supplement: Supplementary file 1 [file vetsci-13-00482-s001.zip › vetsci-4275226-supplementary.pdf]
